# Supplementary material for: Systems Biology Approaches Reveal Potential Phenotype-Modifier Genes in Neurofibromatosis Type 1
Source: Cancers (Basel). 2020 Aug 26;12(9):2416. doi: 10.3390/cancers12092416 (PMC7565824; doi:10.3390/cancers12092416)
Supplement: Supplementary file 1 [file cancers-12-02416-s001.zip › cancers-867098-supplementary.docx]

**Supplementary Materials:**

Systems Biology Approaches Reveal Potential Phenotype Modifier Genes in Neurofibromatosis Type 1


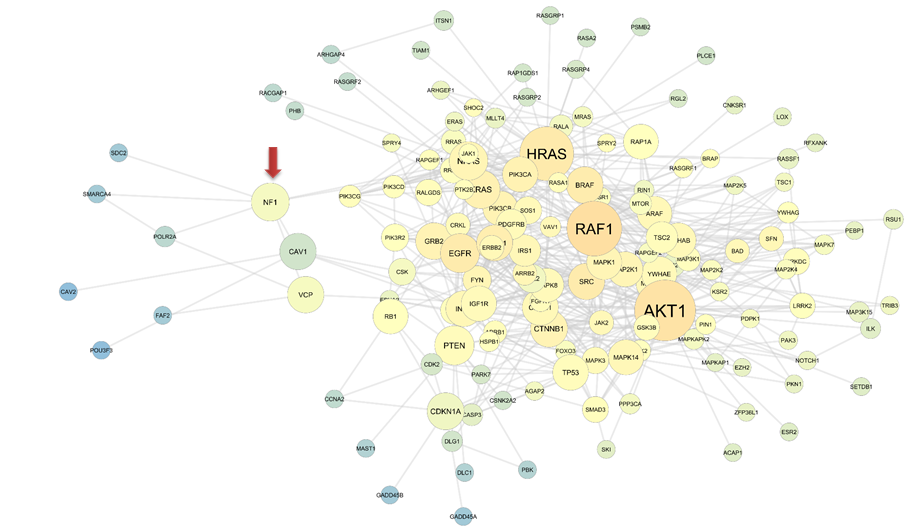


**Figure S1.** Network for GO analysis. Network analysis of the protein-protein interactions (PPI) of the 1449 genes that participate in the selected NF1 gene ontologies. For better visualization, only the first neighbors are presented in the image. The red arrow indicates NF1 in the network. The network was generated using the STRING tool and genes were selected using the Gene Ontology (GO) consortium.


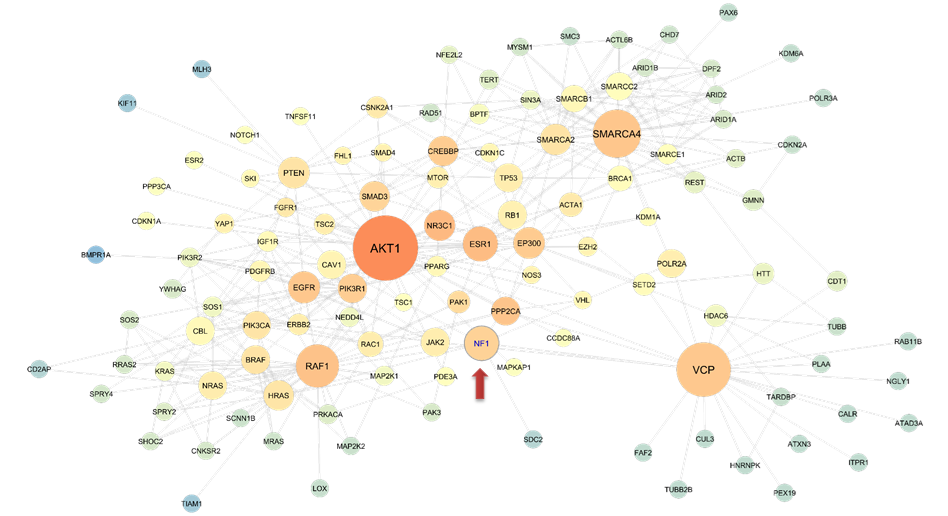


**Figure S2.** Network for HPO analysis**.** Network analysis of the protein-protein interactions (PPI) of the 1697 genes related to neurofibromatosis type 1 phenotypes (OMIM:162200). For better visualization, only the first neighbors are presented in the image. The red arrow indicates NF1 in the network. The network was generated using the STRING tool and genes were selected using the Human Phenotype Ontology (HPO) project.


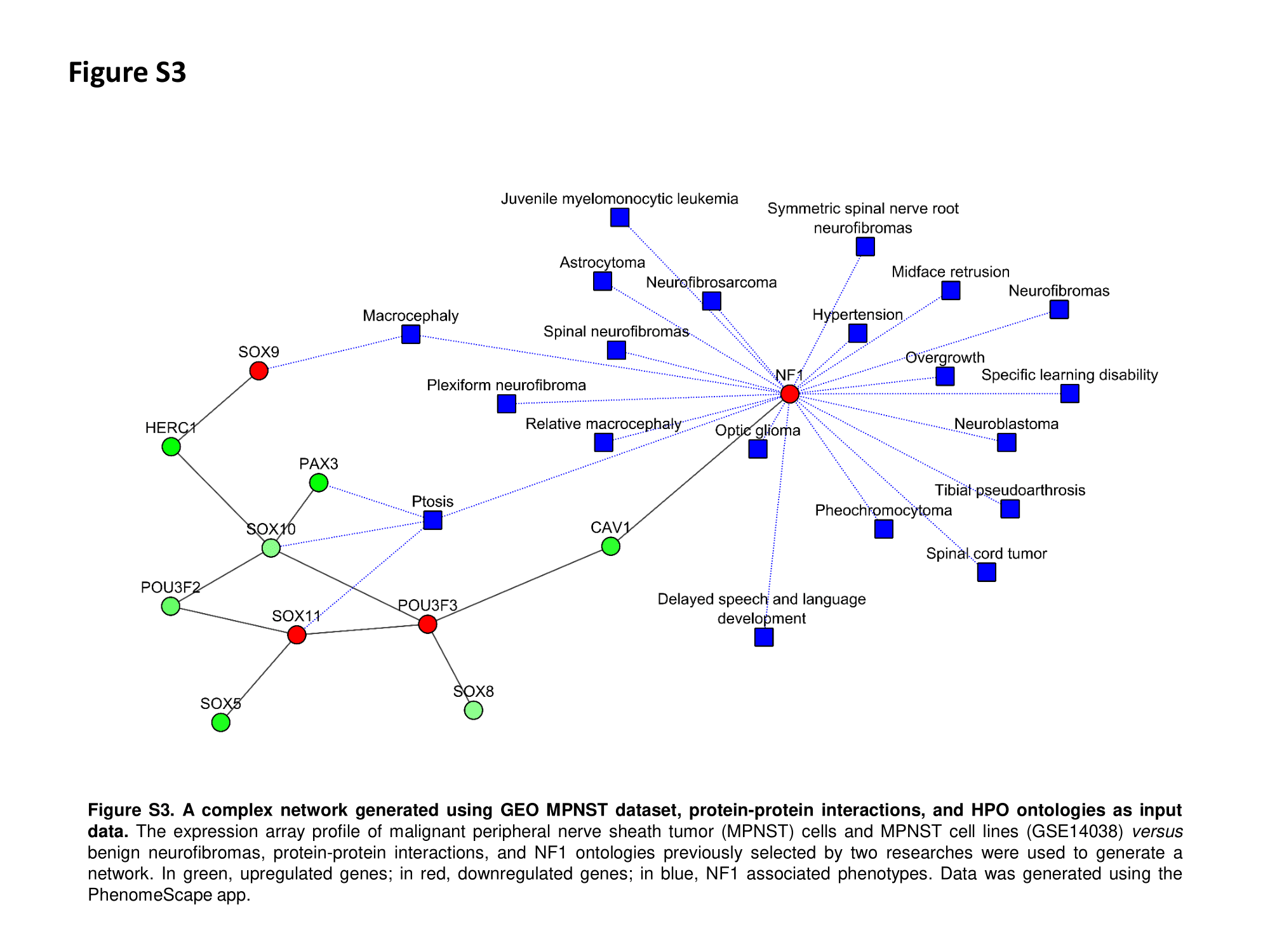


**Figure S3.** A complex network generated using GEO MPNST dataset, protein-protein interactions, and HPO ontologies as input data. The expression array profile of malignant peripheral nerve sheath tumor (MPNST) cells and MPNST cell lines (GSE14038) versus benign neurofibromas, protein-protein interactions, and NF1 ontologies previously selected by two researches were used to generate a network. In green, downregulated genes; in red, upregulated genes; in blue, NF1 associated phenotypes. Data was generated using the PhenomeScape app.


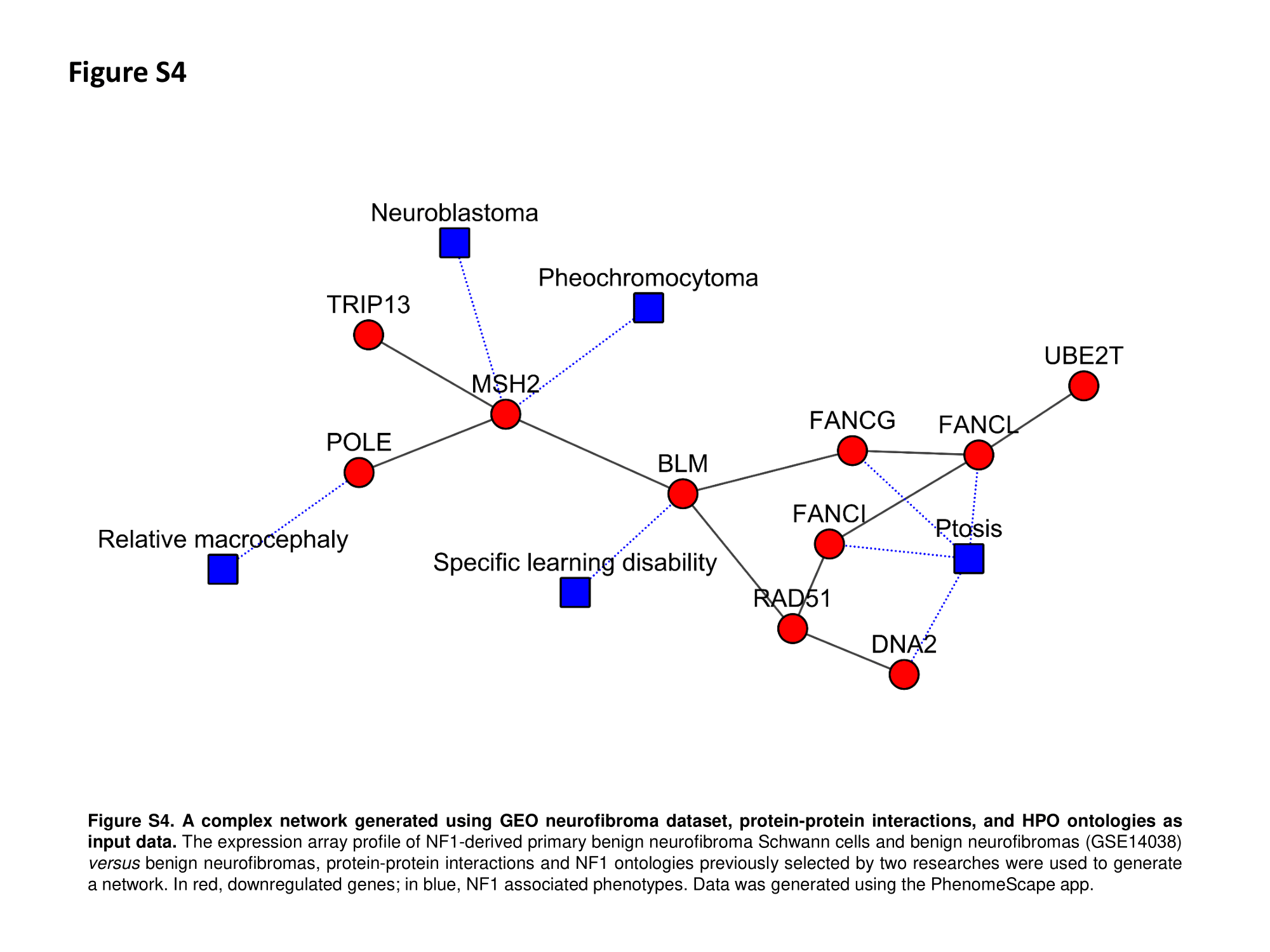


**Figure S4.** A complex network generated using GEO neurofibroma dataset, protein-protein interactions, and HPO ontologies as input data. The expression array profile of NF1-derived primary benign neurofibroma Schwann cells and benign neurofibromas (GSE14038) versus benign neurofibromas, protein-protein interactions and NF1 ontologies previously selected by two researches were used to generate a network. In red, upregulated genes; downregulated genes were not identified in this analysis; in blue, NF1 associated phenotypes. NF1 is absent from the network because its expression was not significantly altered in this analysis. Data was generated using the PhenomeScape app.


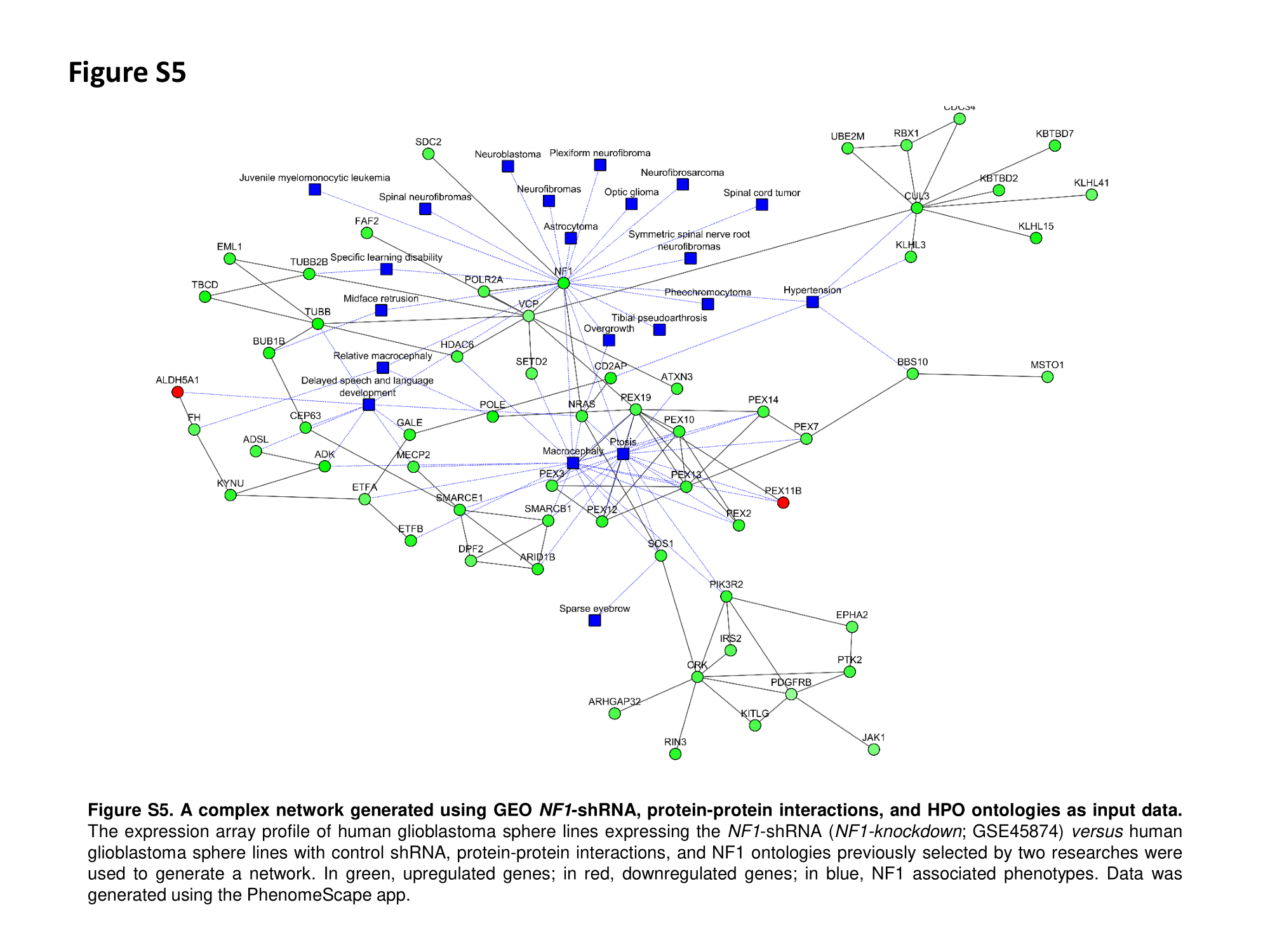


**Figure S5.** A complex network generated using GEO *NF1*-shRNA, protein-protein interactions, and HPO ontologies as input data. The expression array profile of human glioblastoma sphere lines expressing the *NF1*-shRNA (*NF1-knockdown*; GSE45874) versus human glioblastoma sphere lines with control shRNA, protein-protein interactions, and NF1 ontologies previously selected by two researches were used to generate a network. In green, downregulated genes; in red, upregulated genes; in blue, NF1 associated phenotypes. Data was generated using the PhenomeScape app.


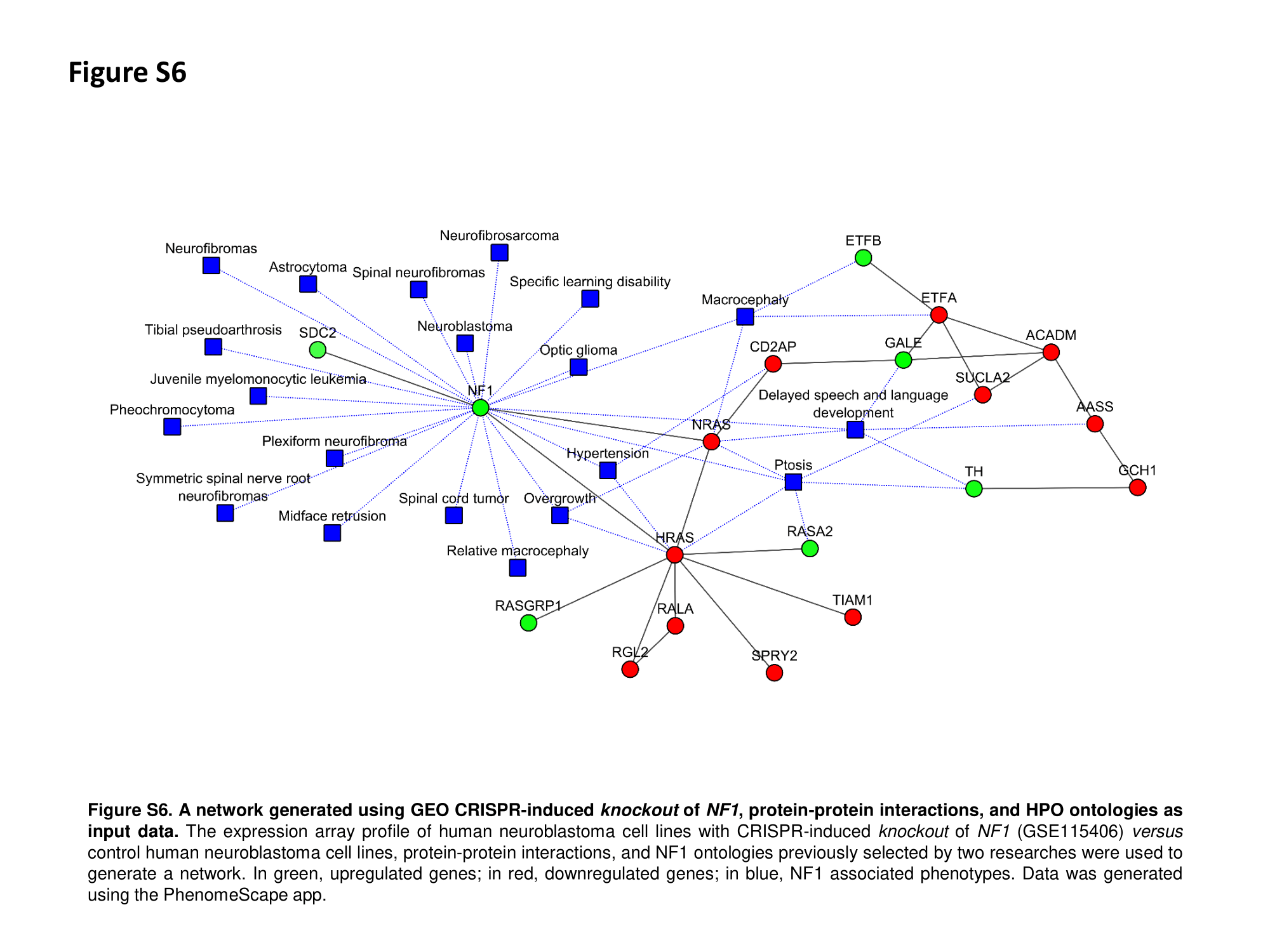


**Figure S6.** A network generated using GEO CRISPR-induced *knockout* of *NF1*, protein-protein interactions, and HPO ontologies as input data. The expression array profile of human neuroblastoma cell lines with CRISPR-induced *knockout* of *NF1* (GSE115406) *versus* control human neuroblastoma cell lines, protein-protein interactions, and NF1 ontologies previously selected by two researches were used to generate a network. In green, downregulated genes; in red, upregulated genes; in blue, NF1 associated phenotypes. Data was generated using the PhenomeScape app.


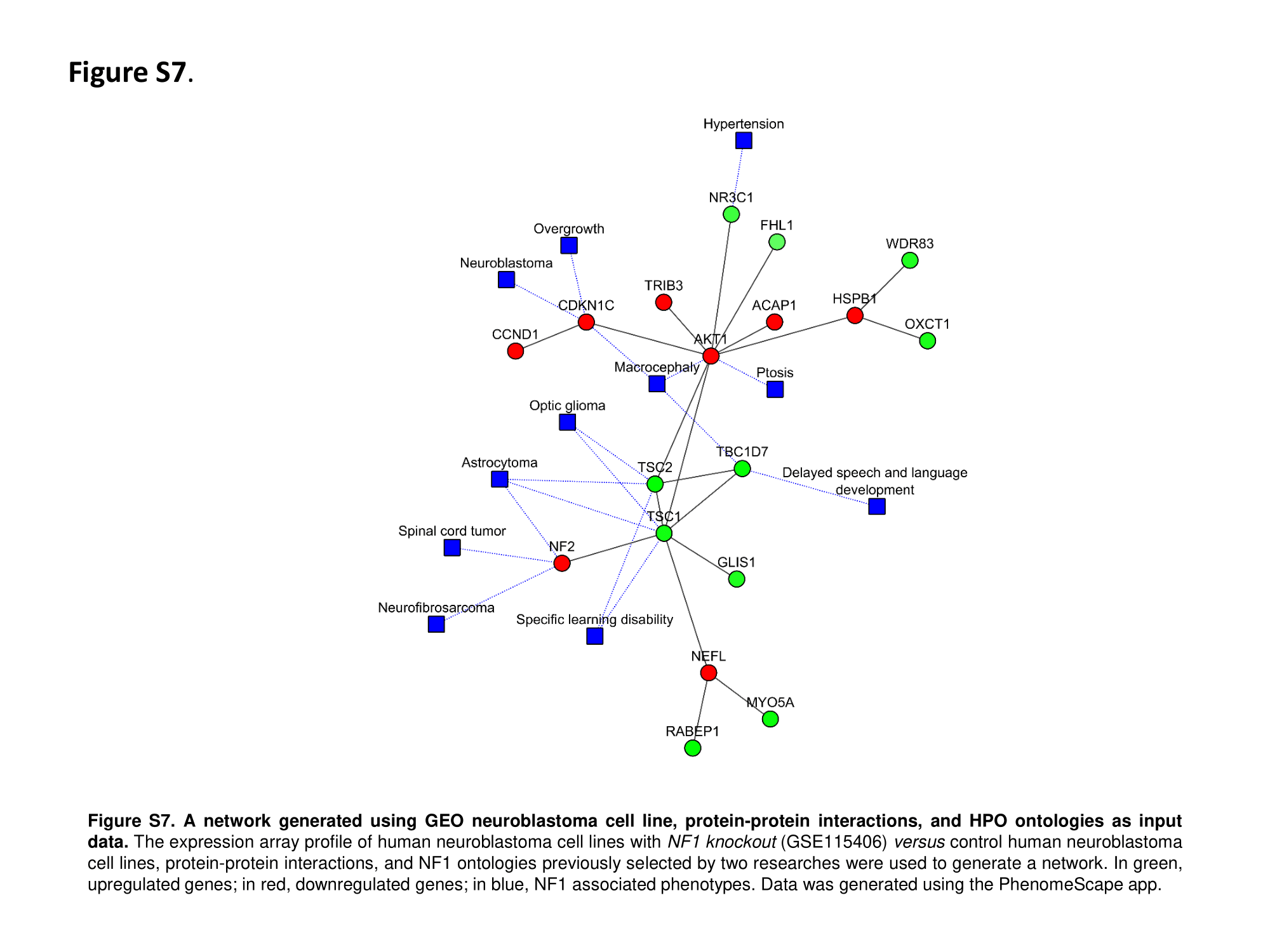


**Figure S7.** A network generated using GEO neuroblastoma cell line, protein-protein interactions, and HPO ontologies as input data. The expression array profile of human neuroblastoma cell lines with *NF1 knockout* (GSE115406) versus control human neuroblastoma cell lines, protein-protein interactions, and NF1 ontologies previously selected by two researches were used to generate a network. In green, downregulated genes; in red, upregulated genes; in blue, NF1 associated phenotypes. NF1 is absent from the network because its expression was not significantly altered in this analysis. Data was generated using the PhenomeScape app.


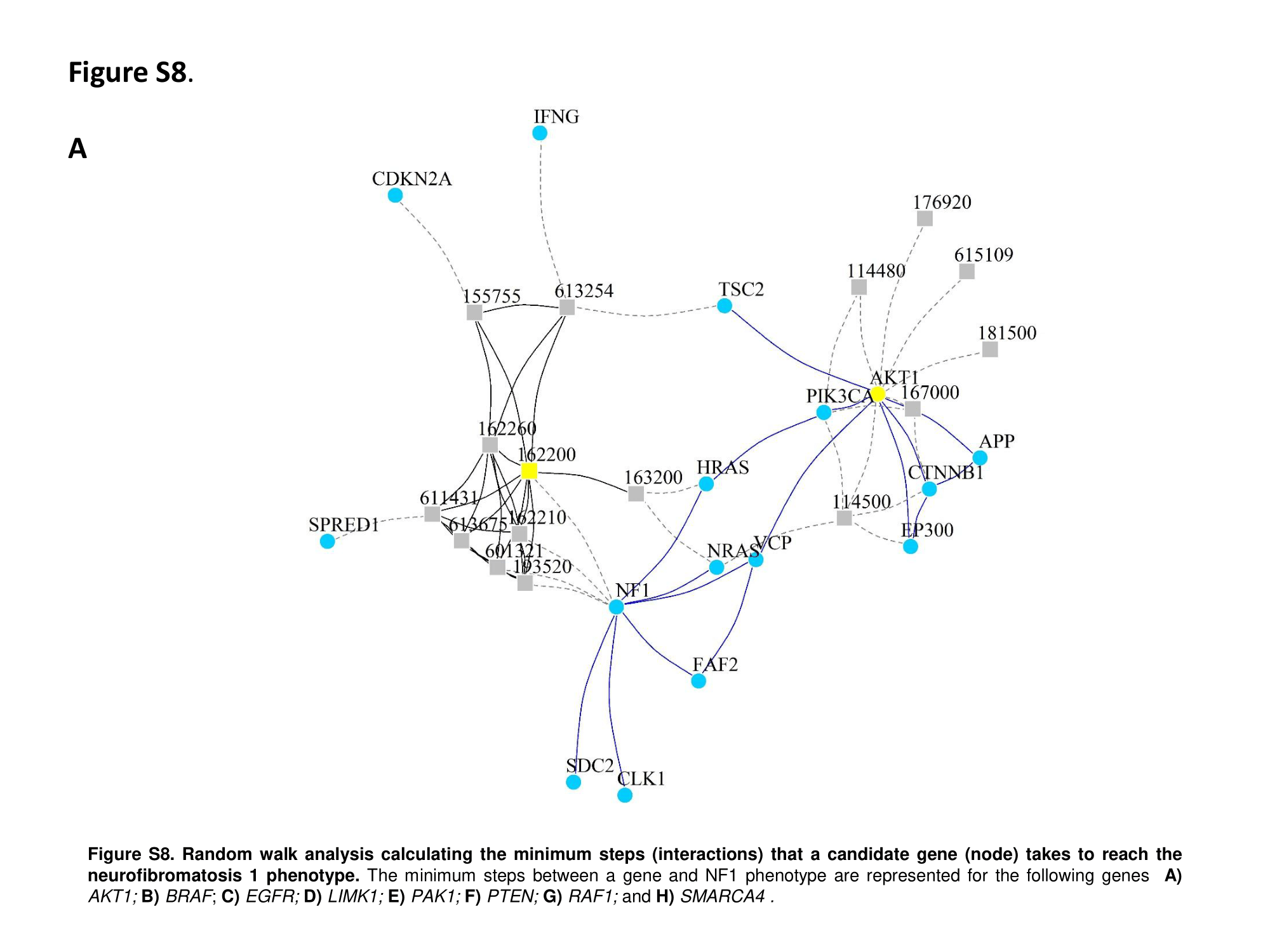

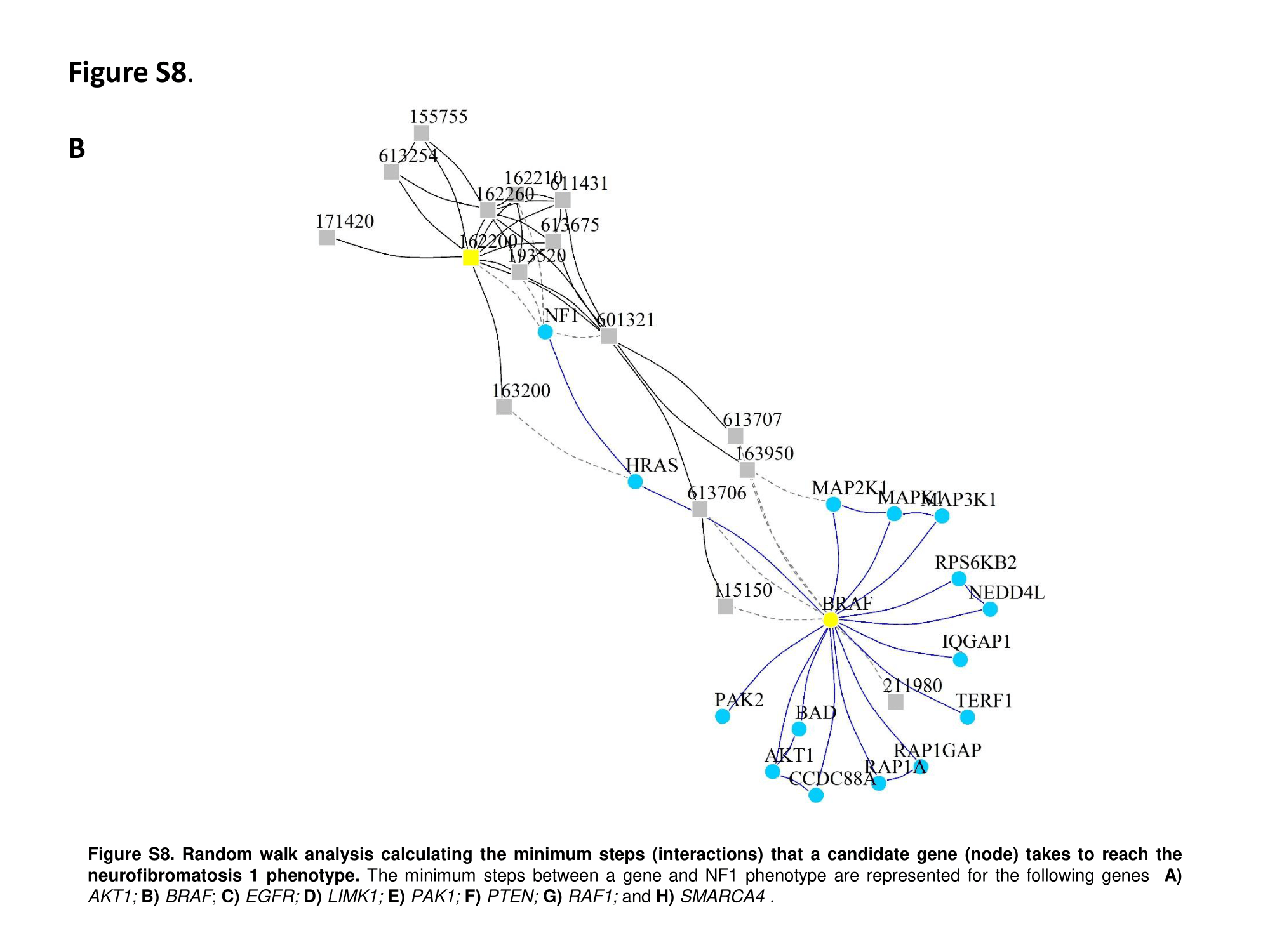

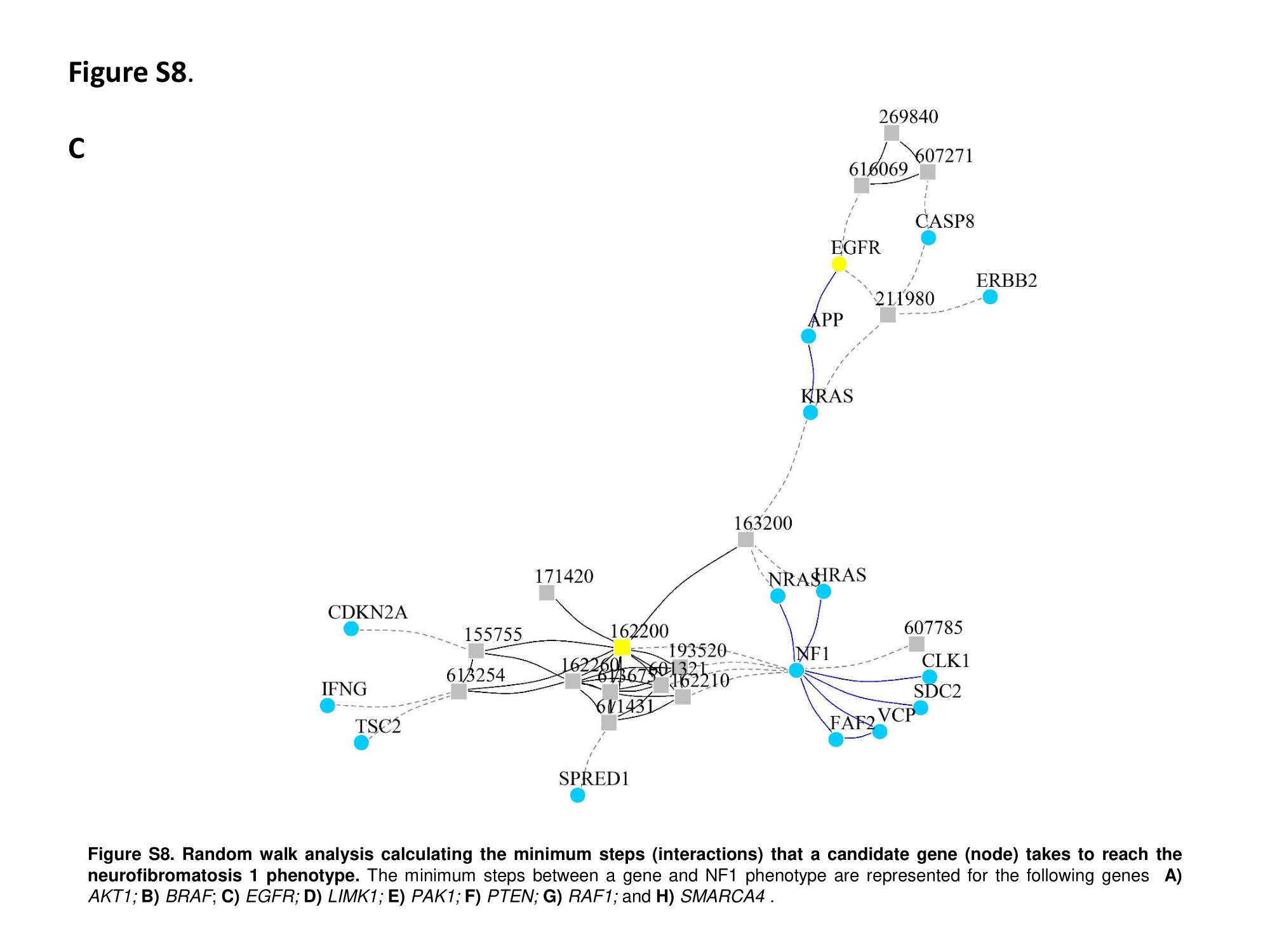

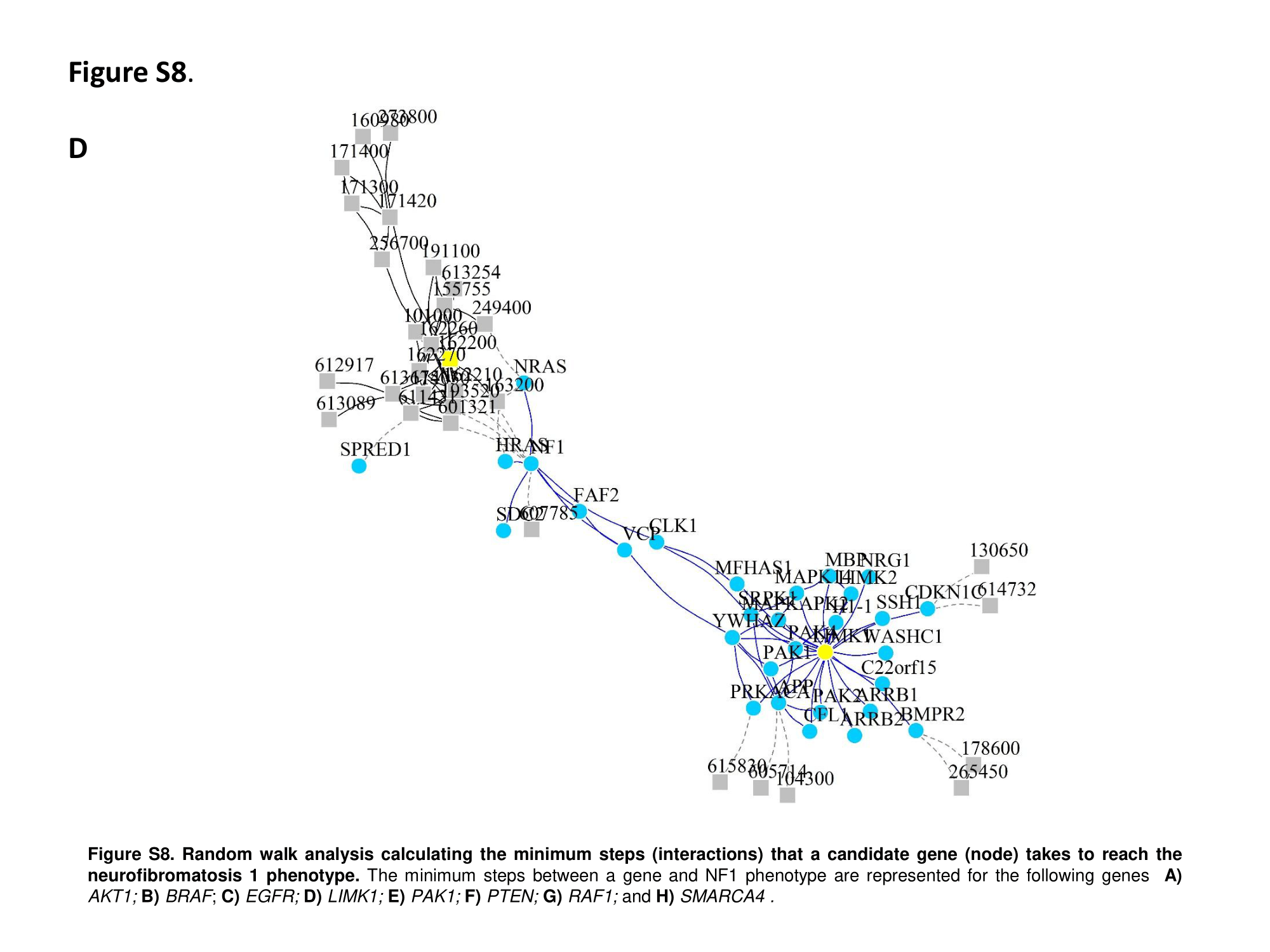

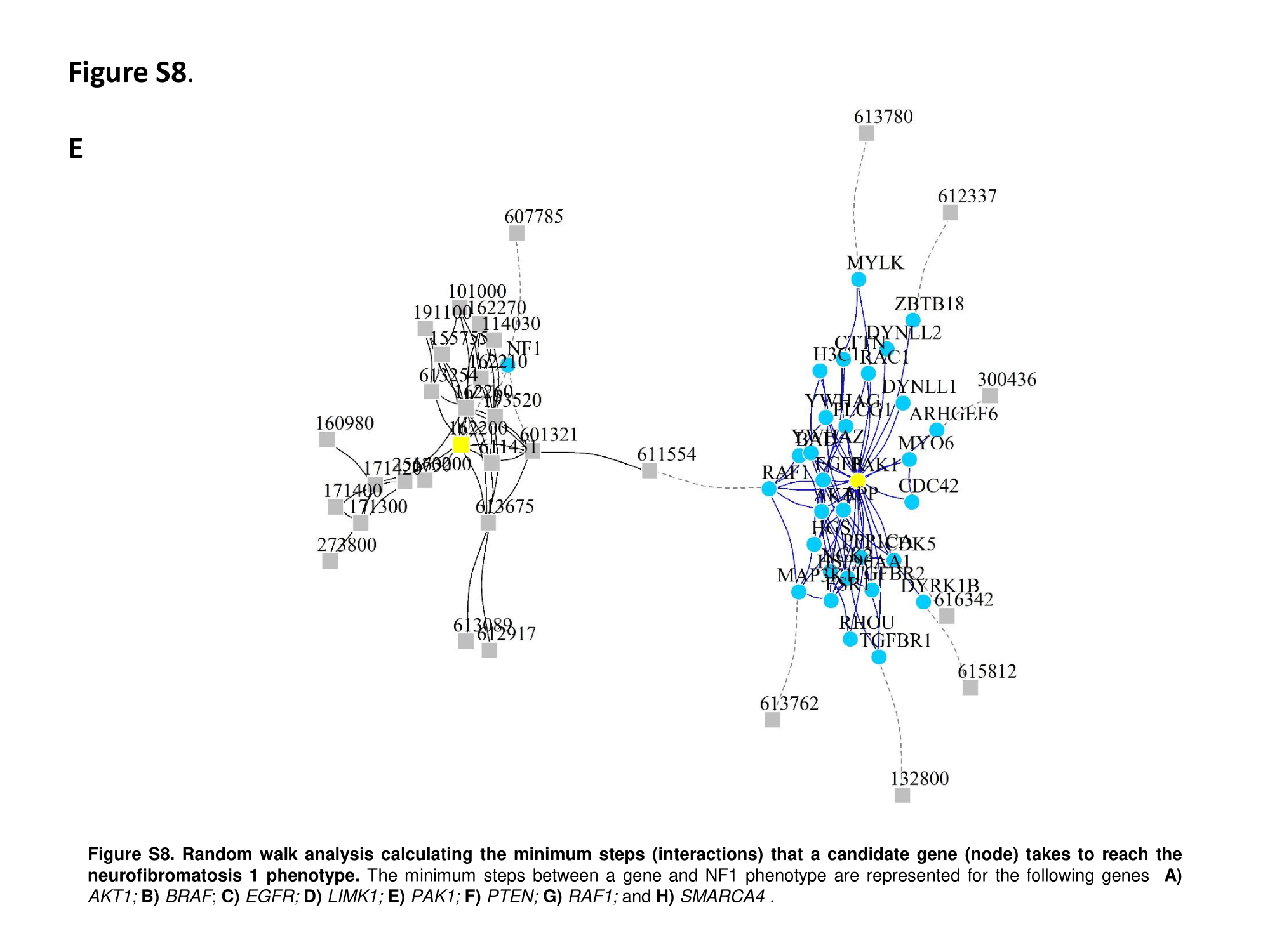

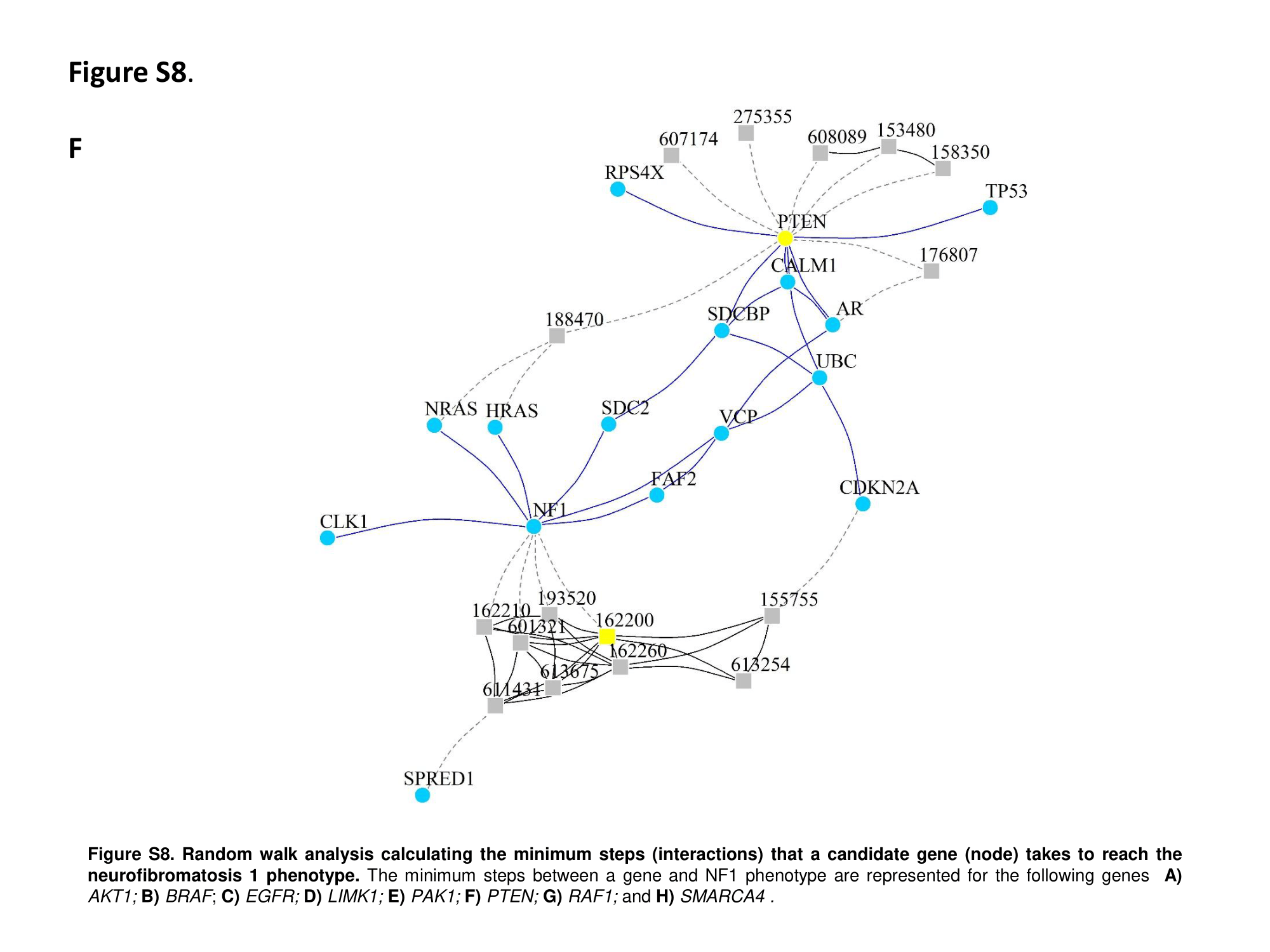

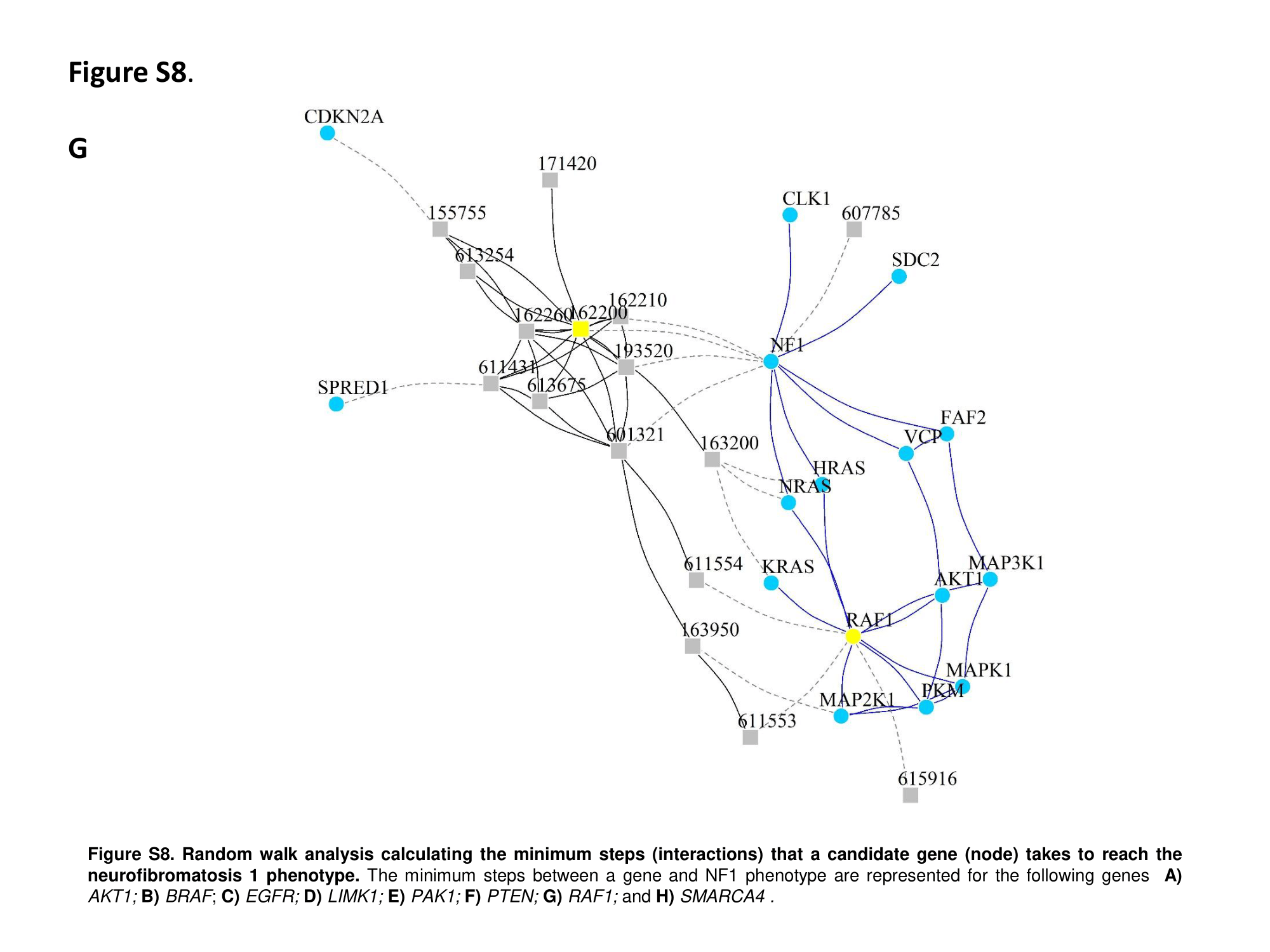

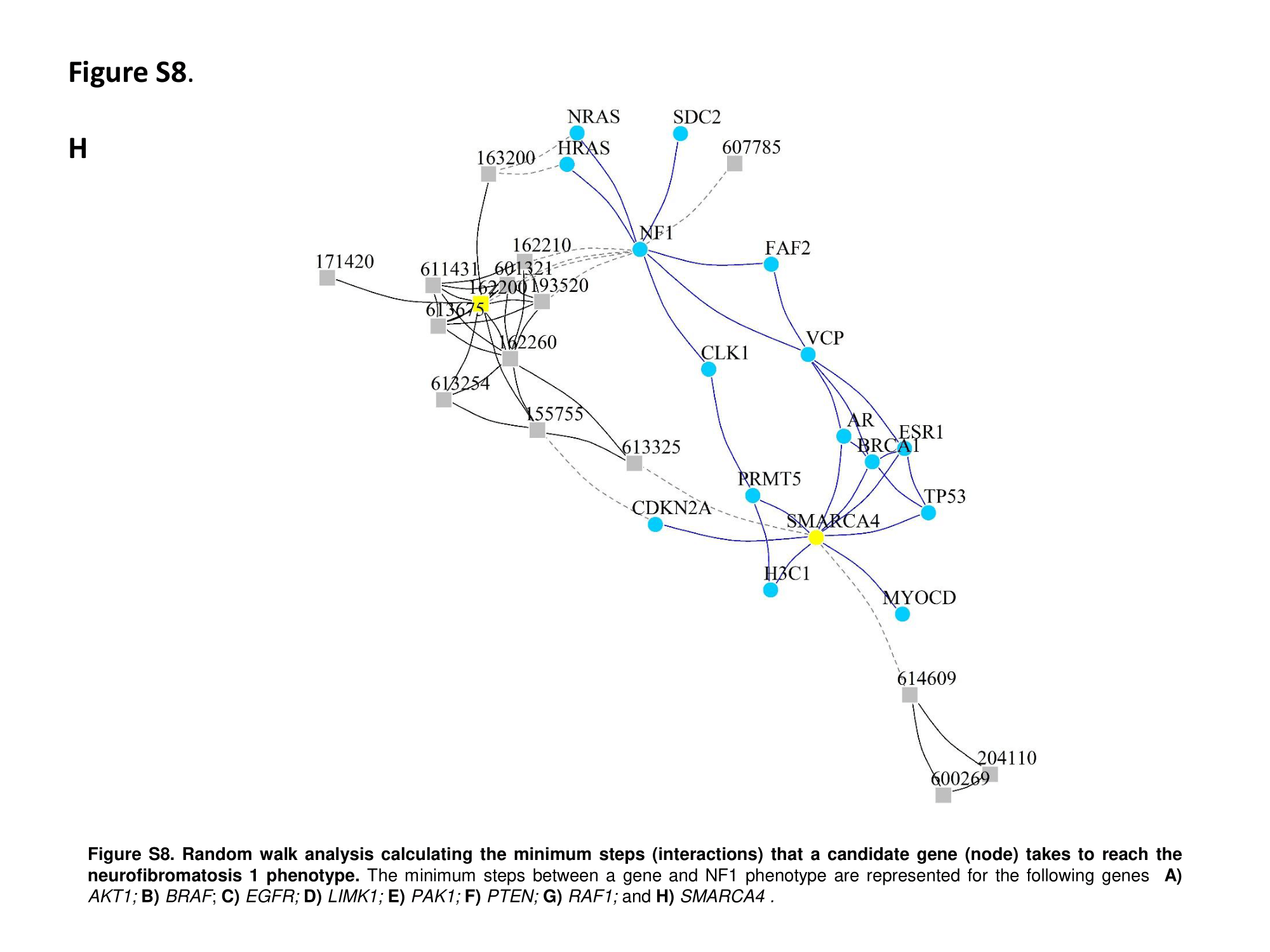


**Figure S8.** Random walk analysis calculating the minimum steps (interactions) that a candidate gene (node) takes to reach the neurofibromatosis 1 phenotype. The minimum steps between a gene and NF1 phenotype is represented for the following genes (**A**) *AKT1;* (**B**) *BRAF*; (**C**) *EGFR;* (**D**) *LIMK1;* (**E**) *PAK1;* (**F**) *PTEN;* (**G**) *RAF1;* and (**H**) *SMARCA4 .*

**Table S1.** Selected NF1 ontologies. Ontologies related to the main phenotypes identified in Neurofibromatosis type 1 selected from the Gene Ontology (GO) and Human Phenotype Ontology (HPO) databases*.

| **Gene Ontology (GO)** | **The Human Phenotype Ontology (HPO)** |
| --- | --- |
| MAPK Cascade [ISS] | Intellectual disability |
| Ras Protein Signal Transduction [ISS] | Sparse and thin eyebrow |
| Schwann Cell Development [ISS] | Neuroblastoma |
| Actin Cytoskeleton Organization [ISS] | Midface retrusion |
| Adrenal Gland Development [ISS] | Global developmental delay |
| Brain Development [ISS] | Overgrowth |
| Cerebral Cortex Development [ISS] | Macrocephaly |
| Cognition [IMP] | Tibial pseudoarthrosis |
| Forebrain Astrocyte Development [ISS] | Specific learning disability |
| Forebrain Morphogenesis [ISS] | Scoliosis |
| Myelination In Peripheral Nervous System [ISS] | Optic nerve glioma |
| Negative Regulation Of MAP Kinase Activity [ISS] | Seizure |
| Negative Regulation Of MAPK Cascade [IMP, ISS] | Hypertrophic cardiomyopathy |
| Negative Regulation Of Ras Protein Signal Transduction [IBA] | Neurofibrosarcoma |
| Negative Regulation Of Neuroblast Proliferation [ISS] | Symmetric spinal nerve root neurofibromas |
| Negative Regulation Of Protein Kinase Activity [ISS] | Plexiform neurofibroma |
| Osteoblast Differentiation [ISS] | Ptosis |
| Peripheral Nervous System Development [ISS] | Astrocytoma |
| Phosphatidylinositol 3-Kinase Signaling [ISS] | Spinal cord tumor |
| Positive Regulation Of Ras GTPase Activity [IDA, IMP, ISS] | Spinal neurofibromas |
| Positive Regulation Of Adenylate Cyclase Activity [ISS] | Neurofibromas |
| Positive Regulation Of Neuron Apoptotic Process [ISS] | Relative macrocephaly |
| Regulation Of Ras GTPase Activity [IMP] | Hypertension |
| Regulation Of Bone Resorption [ISS] | Abnormality of the face |
| Regulation Of Cell-Matrix Adhesion [ISS] | Intellectual disability, mild |
| Regulation Of Glial Cell Differentiation [ISS] | Pheochromocytoma |
| Spinal Cord Development [ISS] | Spinal cord tumor |
|  | Spinal neurofibromas |
|  | Juvenile myelomonocytic leukemia |
|  | Delayed speech and language development |

*All NF1 related ontologies can be found at GO and HPO online databases.

**Table S2.** *NF1* knockdown and knockout assays in GEO database. LogFC and FDR values for the ten potential modifier genes in each NF1 datasets (GSE14038 and GSE115406) selected from GEO database. Statistical significant results are highlighted (>1 or <−1) and a FDR < 0.05).

| **Genes** | **GSE14038** | | **GSE115406** | |
| --- | --- | --- | --- | --- |
|  | **logFC^1^** | **FDR^2^** | **logFC^1^** | **FDR^2^** |
| *AKT1* | −0.020997946 | 0.942409301 | 0.48594508 | 0.004486066 |
| *BRAF* | −0.35120702 | 0.109069372 | −0.23647294 | 0.185829366 |
| *EGFR* | −0.011099513 | 0.945691123 | −0.084229026 | 0.687987305 |
| *LIMK1* | 0.681980811 | 0.004776647 | 0.308737213 | 0.018199507 |
| *NF1* | −0.397118003 | 0.255385186 | −0.290785572 | 0.043400883 |
| *PAK1* | 0.453498463 | 0.028341148 | 0.668222212 | 0.023458709 |
| *PTEN* | −1.162007748 | 0.000000022 | −0.20924434 | 0.059096572 |
| *RAF1* | 0.13797881 | 0.431565535 | 0.044715128 | 0.707227171 |
| *SDC2* | −0.248080524 | 0.592262309 | −0.819968473 | 0.000546736 |
| *SMARCA4* | 0.984439218 | 0.000120502 | −0.288968636 | 0.022068287 |
| *VCP* | 0.146533794 | 0.735016164 | 0.244942227 | 0.020275839 |

^1^ Log fold-change ^2^ False Discovery Rate.

**Table S3.** Tumors with *NF1* nonsense mutations from TCGA database. LogFC and FDR values of the differential expression of the ten potential phenotype modifier genes in the seven types of tumors with nonsense *NF1* mutations selected from TCGA database versus wild-type *NF1* tumors. Statistical significant results are highlighted (>1 or <−1) and a FDR < 0.05).

| **Genes** | **BRCA** | | **GBM** | | **LGG** | | **PCPG** | | **BLCA** | | **CESC** | | **COAD** | |
| --- | --- | --- | --- | --- | --- | --- | --- | --- | --- | --- | --- | --- | --- | --- |
|  | **logFC** | **FDR** | **logFC** | **FDR** | **logFC** | **FDR** | **logFC** | **FDR** | **logFC** | **FDR** | **logFC** | **FDR** | **logFC** | **FDR** |
| *AKT1* | 0.220 | 0.649 | −0.225 | 1 | 0.002 | 1 | 0.564 | 1 | −0.185 | 0.802 | 0.093 | 0.855 | 0.070 | 0.951 |
| *BRAF* | −0.958 | 0.101 | −0.402 | 1 | −0.137 | 0.785 | −0.017 | 1 | −0.086 | 0.972 | 0.197 | 0.784 | 0.040 | 0.989 |
| *EGFR* | −1.618 | 0.371 | −1.855 | 1 | −1.945 | 0.029 | −2.457 | 1 | −0.468 | 0.914 | −1.151 | 0.487 | −1.036 | 0.490 |
| *LIMK1* | 0.039 | 0.961 | 0.047 | 1 | 0.605 | 0.009 | 0.621 | 1 | −0.294 | 0.791 | 0.056 | 0.953 | 0.204 | 0.884 |
| *NF1* | −1.505 | 0.001 | −1.422 | 0.408 | −1.376 | 0.000 | −1.805 | 0.318 | −0.955 | 0.007 | −0.896 | 0.065 | −1.515 | 0.000 |
| *PAK1* | −0.400 | 0.644 | −0.024 | 1 | −0.322 | 0.696 | 0.120 | 1 | 0.284 | 0.837 | −0.164 | 0.807 | −0.262 | 0.750 |
| *PTEN* | −0.297 | 0.556 | 0.299 | 1 | −0.392 | 0.028 | 0.158 | 1 | −0.014 | 1 | −0.007 | 1 | −0.183 | 0.887 |
| *RAF1* | −0.194 | 0.519 | −0.134 | 1 | −0.096 | 0.558 | −0.360 | 1 | −0.075 | 0.971 | −0.028 | 0.977 | −0.086 | 0.911 |
| *SDC2* | 0.537 | 0.519 | −0.134 | 1 | 0.622 | 0.163 | 0.090 | 1 | 0.144 | 0.973 | −1.054 | 0.470 | 0.413 | 0.853 |
| *SMARCA4* | −0.326 | 0.506 | −0.165 | 1 | −0.081 | 0.745 | 0.246 | 1 | 0.040 | 0.986 | 0.373 | 0.451 | −0.151 | 0.904 |
| *VCP* | 0.257 | 0.452 | −0.199 | 1 | 0.149 | 0.200 | 0.281 | 1 | 0.271 | 0.673 | 0.385 | 0.279 | 0.008 | 1 |

BRCA = Breast Invasive Carcinoma; GBM = Glioblastoma Multiforme; LGG = Lower Grade Glioma; PCPG = Pheochromocytoma and Paraganglioma; BLCA = Bladder Urothelial Carcinoma; CESC = Cervical Squamous Cell Carcinoma and Endocervical Adenocarcinoma; COAD = Colon Adenocarcinoma.
